# Determinants of Farmers’ Intention of Straw Recycling: A Comparison Analysis Based on Different Pro-Environmental Publicity Modes

**DOI:** 10.3390/ijerph182111304

**Published:** 2021-10-28

**Authors:** Hao Zhu, Yibin Ao, Hong Xu, Zhongli Zhou, Yan Wang, Linchuan Yang

**Affiliations:** 1 College of Environment and Civil Engineering, Chengdu University of Technology, Chengdu 610059, China; zhuhao1@stu.cdut.edu.cn (H.Z.); xuhong@stu.cdut.edu.cn (H.X.); 2 College of Management Science, Chengdu University of Technology, Chengdu 610059, China; zzl@cdut.edu.cn; 3 Department of Engineering Management, Sichuan College of Architectural Technology, Deyang 618000, China; wangyan_hy09@sina.com; 4 Department of Urban and Rural Planning, School of Architecture and Design, Southwest Jiaotong University, Chengdu 611756, China; yanglc0125@swjtu.edu.cn

**Keywords:** driving factors, straw recycling intention, pro-environmental publicity, theory of planned behavior, social trust, value perception

## Abstract

Promoting the intention of farmers to participate in straw recycling is an effective way to alleviate the contradiction between environmental pollution, scarcity of environmental resources, and sustainable development. In this study, social trust and value perception were integrated into the theory of planned behavior to build a theoretical framework of farmers’ intention to participate in straw recycling, considering the influences of three different pro-environmental publicity modes. A field investigation was used to collect research data in six sample villages. Finally, 761 valid questionnaires were collected, and partial least squares structural equation modeling was applied to test the research hypotheses. The results showed that the influence of attitudes, subjective norms, perceived behavior control, value perception, and social trust on farmers’ straw recycling intentions was different among different pro-environmental publicity modes. Among the three pro-environmental publicity modes, the concentrated pro-environmental publicity mode has the best effect of promoting straw recycling intentions among farmers. This study introduces some targeted suggestions on the aspects of pro-environmental publicity theory and management practice based on the above research results.

## 1. Introduction

Crop straw, as agricultural production waste, is a crucial renewable and low-cost biomass energy with the advantages of high yield and considerable resource potential. According to statistics, the global annual crop of straw is approximately 6 billion tons [1]. However, many developing countries do not maximize the use of straw despite such a large number of straw resources. A large number of straw resources are directly burned, discarded, or buried worldwide, which not only causes waste of resources but also seriously pollutes the ecological environment [2]. Studies have shown that straw burning in the open air leads to serious air pollution [3] and mainly results in the formation of heavy smog in cities [4]. The open burning of straw will also not only cause serious fires [5] but also affects the operation of airports and expressways [6]. Casually discarded or buried straws will lead to soil and water pollution [2]. Some countries (such as Britain and Canada) recycle straw resources and use them for animal husbandry, power generation, fuel, cultivation of mushroom base material, bedding, and bioethanol production [7,8,9]. Thus, turning straw waste into treasure can realize the “win–win” of economy and ecology.

China is a huge agricultural country with an annual crop yield of more than 900 million tons [10], but its average comprehensive utilization rate is only approximately 40% [6]. The Chinese government has implemented a ban on straw burning and taken measures such as returning straw to the field and turning it into fodder, fertilizer, energy, and raw materials; however, straw burning and random discarding still exist [11]. Satellite remote sensing monitoring data of China revealed 1158 straw burning points nationwide in the summer of 2015, and the number of straw burning points in 2019 was 42% lower than that in 2015 [12]. The full recycling of straw has not yet been achieved despite the significant drop in the amount of burned straw.

Straw recycling plays a positive role in promoting the utilization of resources and improving the ecological environment, which belongs to the category of pro-environment behavior [13]. Pro-environment behavior refers to the behavior in which people minimize the negative impact of their activities on the ecological environment and promote the sustainable development of the economy and environment [14]. An increasing number of researchers have begun to focus on pro-environment behavior with the rising aggravation of environmental problems. For example, Wang et al. [15] used the theory of planned behavior to explore the influencing factors of e-waste recycling intention and found that attitude, subjective norms, perceived behavior control, and economic motivation had significant positive effects on e-waste recycling intention. Meng et al. [16] found that publicity and education, accessibility of facilities, intention to participate in waste sorting, and pro-environmental awareness of residents are the main factors affecting the waste sorting and recycling behavior of urban residents. Yuan et al. [17] suggested that perceived behavioral control had the largest influence on the intention of the public to participate in urban green space governance. Jia et al. [18] showed that attitude, perceived behavior control, and personal norms could positively influence the energy-saving behavior of student dormitories, while subjective norms have no significant influence. However, compared with other pro-environment behaviors, relevant studies on the driving factors of farmers’ straw recycling intention are few.

Farmers are the main body of rural economic activities. The cognition, attitude, and straw recycling intention of farmers not only determine the utilization of local straw resources and efficiency but also have a profound impact on the rural ecological environment. Therefore, enhancing the straw recycling intention of farmers is of considerable significance for alleviating the shortage of rural resources and energy, curbing the deterioration of the rural ecological environment, and improving the efficiency of straw recycling in rural areas. As a rational economic man, the straw recycling behavior of farmers is affected by costs and benefits. Studies have shown that farmers’ perception of the value of straw resource utilization can positively affect their adoption behavior of straw returning technology [19]. Studies have also found that a strong perception of the economic value of straw indicates the increased willingness of farmers to participate in the straw market circulation [20]. As farmers in the social network structure, their straw disposal behavior is not an independent economic decision-making process but will be affected by social trust with interpersonal interaction attributes [21]. Studies have shown that social trust promotes environmental public governance and has an important impact on the pro-environmental behavior of farmers. For example, He et al. [22] demonstrated that social trust plays a significant promoting role in the decision-making of agricultural waste resource utilization by farmers. Li et al. [23] reported that social trust had a significant impact on the adoption behavior of soil testing formula fertilization technology and fertilizer reduction and use behavior of farmers. In addition, publicity can influence the cognition of things of an individual to a certain extent [24]. Pro-environment awareness of farmers and the value of straw recycling must be improved through publicity [25]. Studies have confirmed that the straw recycling behavior of farmers will be affected by publicity [25]. The publicity of protecting the ecological environment can effectively improve the pro-environmental awareness of residents, which is conducive to their active participation in pro-environmental behavior [26,27].

Although existing studies have confirmed the applicability of the theory of planned behavior in pro-environment behavior, its applicability in specific straw recycling behavior of farmers remains to be tested. In addition, the current research confirms the importance of environmental publicity on the environmental behavior of residents but does not delve into the specific impact of specific pro-environmental publicity modes.

This study takes Jintang County in Chengdu City as an example to further the following exploration to fill the aforementioned research gaps. (1) The theory of planned behavior was extended considering the value perception and social trust of straw recycling to build a driving factor model of farmers’ straw recycling intention. (2) Three different pro-environmental publicity modes were designed to collect research data, and the different influences of driving factors on the straw recycling intentions of farmers were analyzed under different pro-environmental publicity modes. The rest of this paper includes the theoretical basis and research hypotheses, methods, results, discussions, and conclusions.

## 2. Theoretical Framework and Research Hypothesis

The theory of planned behavior (TPB), which is developed from the reasoned action (TRA) theory, is widely used to predict the pro-environmental behavior of individuals [28]. TRA solely addresses volitional control and does not consider non-volitional factors such as opportunities and resources necessary to perform an act [29]. Therefore, Ajzen (1991) [28] extends the perceived behavior control (PBC) on the basis of TRA and develops the TPB. Perceived behavior control (PBC) is the perceived difficulty of behavior [28], reflecting past experience and expected barriers [24]. TPB regards human behavior as the result of careful consideration and planning.

TPB holds that the intention to act is the only reliable psychological determinant of actual behavior. Moreover, attitude (A), subjective norms (SNs), and perceived behavioral control (PBC) are the intention antecedents [28]. Attitude refers to a positive or negative evaluation of behavioral consequences [28]. Subjective norms reflect the perceived social pressure to engage in the behavior, while perceived behavioral control is related to the individual’s perception of his/her ability to execute the behavior [28]. TPB is often used to study the environmental behaviors of farmers, such as their ecological breeding behavior [30], ecological planting behavior [31], agricultural production waste disposal behavior [32], water-saving behavior [33], implementation of non-subsidized agricultural pro-environmental measures [34], intention to protect wetlands [35], agricultural and forestry innovation behavior [36], and non-point source pollution control [37]. However, studies on the application of TPB to straw recycling are few. Straw recycling behavior is a planned behavioral decision of farmers, which theoretically follows the TPB. The TPB believes that the behavioral intention will be strong when people have a positive attitude toward a certain behavior. Moreover, positive subjective norms for a certain behavior strengthen the behavioral intention of people. Favorable conditions and few obstacles will enhance the intention of people to conduct the behavior [28]. Derived from TPB, the following hypotheses are proposed in this study.

**Hypothesis** **1** **(H1).** *The farmers’ positive **attitude** toward straw recycling can significantly positively affect their **intention** towards straw recycling.*

**Hypothesis** **2** **(H2).** *The farmers’ **subjective norms** of straw recycling can significantly positively affect their **intention** towards straw recycling.*

**Hypothesis** **3** **(H3).** *The farmers’ **perceived behavioral control** of straw recycling can significantly positively affect their **intention** towards straw recycling.*

The explanatory degree of TPB for individual specific behavior is limited, and the explanatory power can be enhanced by adding other factors [38,39]. The countryside is a society of acquaintances. Farmers’ trust in their relatives, neighbors, and village cadres affects their daily behavior [40]. In addition, rational farmers’ behavioral choices are usually value-oriented. Therefore, this study incorporates social trust and value perception into the TPB framework.

Sociologists believe that social trust (ST) is an important kind of social capital [41]. Social trust, which is the product of social–cultural norms and institutions, can be divided into interpersonal and institutional trust according to the objects of trust [42,43]. Interpersonal trust takes the emotion between people as a bond and often occurs in the primary group (such as family members) and the secondary group (such as neighbors) [44]. This type of trust has the characteristics of closeness and distance, which also leads to differences in the strength of trust. Institutional trust often depends on legal, political, and other institutional environments and is caused by social phenomena based on “non-interpersonal” relationships. Institutional trust will become an important mechanism with the progress of society. Trust in village cadres can also be regarded as institutional trust [22]. Existing studies have shown that social trust has a remarkable promoting effect on public environmental governance behavior and has a significant positive correlation with environmental behavior [45,46].

Value perception (VP) is an important factor affecting the decision behavior of farmers. A strong value perception of farmers on green agricultural production will strengthen their enthusiasm to participate in such a production [47]. The value perception in this study is the subjective judgment of the farmer on the value of straw recycling based on himself/herself. A rational decision of a farmer will follow the principle of profit maximization. Different farmers have different perceptions of straw recycling value, and their intention of straw recycling is different [20]. Therefore, the following hypotheses are proposed in this study.

**Hypothesis** **4** **(H4).** *The **social trust** of farmers can significantly and positively affect their **intention** towards straw recycling.*

**Hypothesis** **5** **(H5).** *The farmers’ **value perception** of straw recycling can significantly positively affect their **intention** towards straw recycling.*

Sociologists believe that individual psychological factors can be influenced to some extent by the spread of information [48]. The dissemination of information will enable residents to obtain certain aspects of knowledge. According to our previous field research in rural areas, there are differences in publicity modes in different places, which lead to differences in farmers’ intentions and behaviors. Therefore, on the basis of previous field research findings, this study further explored the differences in different pro-environmental publicity modes on farmers’ straw recycling intentions and put forward the following hypothesis:

**Hypothesis** **6** **(H6).** *The influence of attitudes, subjective norms, perceived behavioral control, social trust, and value perception on recycling intention is different among the three pro-environmental publicity modes.*

Based on the existing literature, this study further explored the influence of value perception and social trust on farmers’ straw recycling intentions and integrated value perception and social trust into TPB, enriching the research content of rural straw management. In addition, this study fills the research gap on the difference in the straw recycling intentions of farmers under different pro-environmental publicity modes.

**Figure 1:**
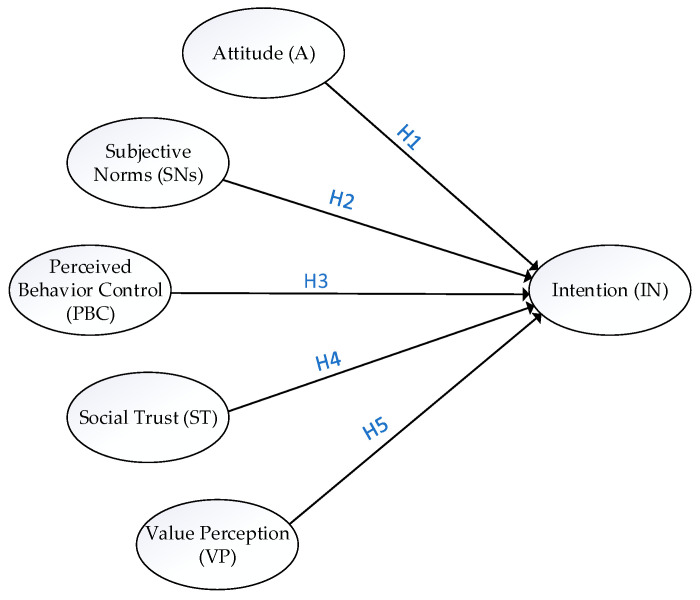
Driving factors of the straw recycling intentions of farmers.

The theoretical driving factor framework of the straw recycling intentions of farmers is proposed on the basis of the hypothesis above, as shown in Figure 1.

## 3. Methodology

### 3.1. Questionnaire Design

The questionnaire of this study mainly contains two parts: (1) basic information of interviewees, including gender, age, education level, and personal annual income [49,50]; (2) items of driving factors (as shown in Table 1), including social trust [51], value perception [16], attitude [15], subjective norm [15], perceived behavioral control [16], and intention [15]. The items of driving factors were measured by a Likert five-point scale (1 means completely disagree, and 5 means completely agree) [52]. Notably, the items of perceived behavioral control describe the farmers’ perception of the time and energy spent on straw recycling. The reverse scoring revealed that a high score indicates minimal time and energy spent by farmers considering straw recycling.

### 3.2. Sample Selection

This study selected Jintang County in Chengdu City as the research site. First, Jintang County has good geographical conditions and is located in the heart and belly of Chengdu Plain, with a mild climate and good light and temperature conditions, which can fully meet the needs of crop growth with double cropping yearly. Second, the agricultural production conditions are remarkably superior. Jintang County has established eight modern agricultural demonstration parks, forming a certain scale of vegetables, fruits, edible fungi, and other production bases. Third, Jintang County has a policy background of the comprehensive utilization of straw. In 2017, Chengdu City issued the “Action Plan for the Pilot Comprehensive Utilization of Straw in Sichuan Province (2017–2020)”, which listed Jintang County as one of the pilot counties to conduct the comprehensive utilization of straw in Chengdu. Fourth, straw recycling is a real problem that must be urgently solved in Jintang County. In July 2020, the researchers visited Jintang County for the first time and learned that the straw pollution problem in this county restricted the development of the local tourism industry, thus becoming the most urgent problem for local village officials and farmers to solve.

Therefore, this study selects two sample villages in plain, hilly, and construction areas based on the local geographical situation and after discussion with local village cadres: Ronghua and Shuangyan Villages in the plain area; Hongqi and Shuangxin Villages in the hilly area; and Zhongfu and Shuangjiang Communities in the construction area. The information of the sample villages is shown in Table 2.

### 3.3. Pro-Environmental Publicity Modes and Data Collection

The pro-environmental publicity of this study means that the transmitter disseminates information related to environmental protection through various means to make farmers aware of the seriousness of environmental pollution and trigger farmers to participate in straw recycling and environmental protection activities. The publicity mode refers to the way and channel of spreading information, and there are many kinds of environmental publicity modes. After communication with the local government, the researchers designed four environmental protection publicity modes for farmers to explore the difference in straw recycling intention of farmers under the following different modes. (1) Non-intervention mode (Mode 1): Without any administrative instructions and publicity means, residents were directly randomly selected to conduct a questionnaire survey and collect research data. (2) Concentrated pro-environmental publicity mode (Mode 2): The researchers used group meetings to perform concentrated pro-environmental publicity activities for randomly selected farmers, and the questionnaire was filled out by the selected farmers during the group meeting. (3) One-on-one and face-to-face pro-environmental publicity mode (Mode 3): With the support of village cadres, researchers randomly selected farmers to conduct one-on-one and face-to-face pro-environmental publicity activities, and then the farmers completed the questionnaire with the help of the researchers. (4) Network diffusion model of pro-environmental publicity mode (Mode 4): A five-minute pro-environmental publicity video was made by the researchers in advance. During the survey, the researchers sent the video through WeChat to the randomly selected farmers, who watched the video before completing the questionnaire. However, the research team ultimately did not use the fourth mode due to the limitations of the network signals in rural areas and the non-smartphones of farmers. This study finally adopted the first three modes of pro-environmental publicity to investigate and collect research data.

**Figure 2:**
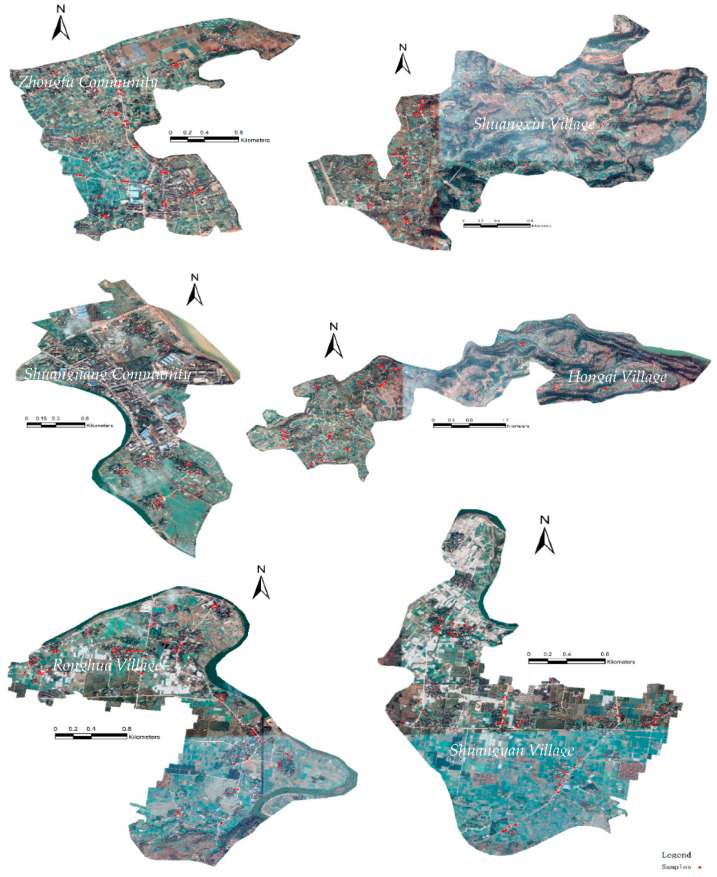
Location distribution map of respondents.

A total of 4–13 people from each village group were selected to participate in the survey during the process of pro-environmental publicity and data collection (in September 2020) to present representative data. A total of 786 questionnaires were collected under the three pro-environmental publicity, in which 25 invalid ones were excluded and 761 valid ones were finally obtained, with an effective rate of 96.8%. Among these questionnaires, 128, 133, and 500 were collected in Mode 1, Mode 2, and Mode 3, respectively. The survey personnel conducted spatial positioning of the surveyed households to ensure the uniform spatial distribution of the sampled farmers. The spatial distribution of the respondents is shown in Figure 2. The valid respondents included more women (53.5%) than men (46.5%). Most of the respondents were over 40 years old because most young people go out for work. The annual family income was between 20,000 and 30,000 yuan (42.0%), followed by less than 20,000 yuan (30.0%). The majority of respondents received primary education (44.8%), followed by junior secondary education (28.8%). Detailed information of the interviewees is shown in Table 3.

Harman’s single-factor test was used in this study to test the common method bias. The analysis results showed that several factors were extracted, and the largest factor accounted for 26.534% (<40%) of the total covariance. Thus, common bias was not a critical issue.

### 3.4. Statistical Tool

SPSS24.0 (IBM, New York, NY, America) was used in this study to sort out the survey data and compare the differences in participation intention of respondents in straw recycling. Structural equation models are widely used to estimate direct and indirect causality between multiple variables [53]. Multiple group partial least squares structural equation model (PLS-SEM) of the software Smart PLS 3.3.3 (SmartPLS GmbH, Boenningstedt, Ahornstrasse, Germany) was used to fit the theoretical model. The structural equation model is developed on the basis of statistical theory, which is the comprehensive application and improvement of statistical methods such as exploratory factor analysis, confirmatory factor analysis, model path analysis, multiple regression analysis, and variance analysis. PLS-SEM can minimize the residual differences of endogenous variables and clarify the complex relationships among multiple variables at the same time. Compared with the covariance method, PLS-SEM is suitable for testing the significance of the hypothesis path because of its high prediction accuracy and low sensitivity to the normality problem [54]. In this study, the data were evaluated by external and internal measurements, followed by hypothesis testing [55]. The outer model measurement involves evaluating the reliability and validity of the data. After the outer model measurement was qualified, the internal model measurement was further used to test the research hypothesis.

## 4. Results

### 4.1. Difference Analysis of Variable Scores

**Figure 3:**
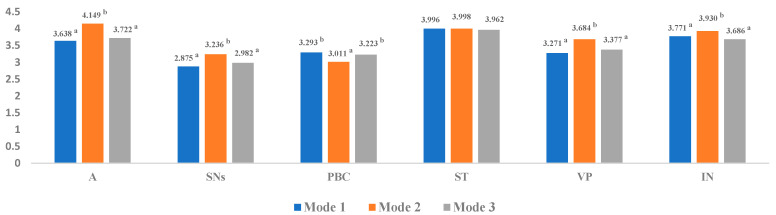
Differences of each variable under different pro-environmental publicity modes. Note: In each variable, no significant difference is found between the two groups of values with the same superscript, the two groups with different superscripts have significant differences, and the group of superscript ^b^ is larger than that of superscript ^a^.

The data could not strictly conform to the normal distribution considering the subjectivity of questionnaire selection of farmers; thus, the rank-sum test was suitable. Therefore, the Kruskal–Wallis test was used in this study to determine differences in the straw recycling intentions of farmers and their driving factors under different pro-environmental publicity modes [56]. The test results are shown in Figure 3. Attitude, subjective norms, value perception, and intention have significant differences among the three pro-environmental publicity modes. Specifically, attitude, subjective norms, value perception, and intention in Mode 2 are significantly higher than those in Mode 1 and Mode 3. No significant difference was found in social trust among the three pro-environmental publicity modes. Interestingly, the perceived behavioral control of Mode 2 was significantly lower than that of Mode 1 and Mode 3, suggesting that farmers in Mode 2 believed that straw recycling would consume considerable amounts of time and effort.

### 4.2. Difference Analysis of Path Coefficients

#### 4.2.1. Measurement Model Analysis

Reliability refers to the internal consistency between indicators, which can be verified by Cronbach’s Alpha (≥0.5) [57], composite reliability (CR) (≥0.7) [58], and factor load (≥0.5) [58]. The test results of this study show that the Cronbach’s α distribution of all variables ranges from 0.630 to 0.949 (Table 3), which are all larger than 0.5. The CR of each variable ranged from 0.784 to 0.975, which is larger than 0.7 (Table 4). Meanwhile, the factor load distribution range of each item is from 0.548 to 0.980 (Table 1). Overall, the reliability of the measurement model is acceptable.

Validity is used to measure the effectiveness of a given structure, including convergence and discriminative validity. Convergent validity is evaluated by the average variance extracted (AVE), and an AVE larger than 0.5 indicates that the convergent validity of the structure is appropriate [59]. The AVE of each variable in this study ranges from 0.556 to 0.951 (the full results are shown in Table 4), which is larger than 0.5. Discriminant validity is used to determine the degree of differentiation between different variables. Table 4 shows that the inter-variable correlations were lower than the square root of the AVE of that variable. Therefore, the measurement model passed the validity test.

#### 4.2.2. Explanatory Power of the Structural Model

The explanatory capability of the structural model is further analyzed on the basis of the qualified measurement model. The R^2^ of the endogenous structure can measure the explanatory power of the model. The R^2^ of IN is 0.247, and an R^2^ larger than 0.1 indicates that the explanatory power of the model is acceptable [42]. The overall goodness of fit (GoF) index can be calculated by the formula (GoF = (Ave × R^2^) ∙ 1/2), which is used to measure the fitting degree of the model [60]. Prior research indicates that the model fit degree is small, medium, and large when the calculated GoF is larger than 0.1, 0.25, and 0.36, respectively [61]. The GoF value of this study is 0.425, indicating that the model fits the data well.

#### 4.2.3. Multi-Group Analysis

Multi-group analysis [62] is often used to compare the differences in path coefficients between different populations [63,64]. Therefore, after the verification of the structural model is qualified, multi-group analysis is adopted to further explore the differences in the impact of value perception, social trust, attitude, subjective norms, and perceived behavioral control on the intention of different pro-environment publicity modes.

**Figure 4:**
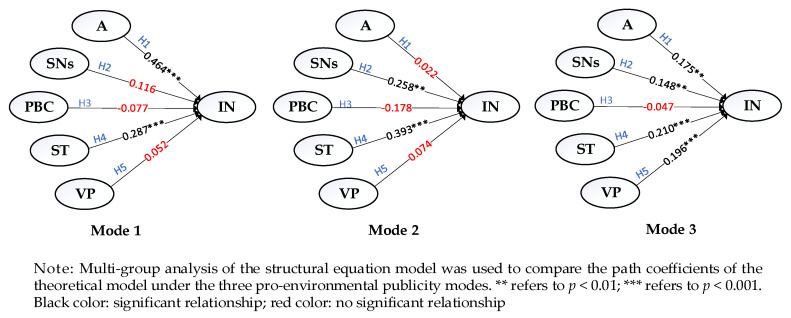
Path coefficient in the three pro-environmental publicity modes.

The significance of the structural path coefficients of each model was illustrated by the t-value calculated by bootstrapping (5000 subsamples). The T-statistic larger than 1.96 (significant at the level of 5%) was selected as the threshold in this study [65], and the analysis results are shown in Figure 4. The results are described as follows.

(1) Attitude has a significant positive effect on intention (H1). The hypotheses (H1) in Mode 1 (*β* = 0.464, *p* < 0.001) and Mode 3 (*β* = 0.175, *p* < 0.01) are supported, but those in Mode 2 (*β* = 0.022, *p* > 0.05) are unsupported.
(2) Subjective norms have a significant positive effect on intention (H2). The hypotheses (H2) in Mode 2 (*β* = 0.258, *p* < 0.01) and Mode 3 (*β* = 0.148, *p* < 0.01) are supported, but those in Mode 1 (β = 0.116, *p* > 0.05) are unsupported.
(3) Perceived behavioral control has a significant positive effect on intention (H3), and the hypotheses (H3) in Modes 1, 2, and 3 are unsupported.
(4) Social trust has a significant positive effect on intention (H4), and the hypotheses (H4) in Mode 1 (*β* = 0.287, *p* < 0.001), Mode 2 (*β* = 0.393, *p* < 0.001), and Mode 3 (*β* = 0.210, *p* < 0.001) are all supported.
(5) Value perception has a significant positive effect on intention (H5). The hypotheses (H5) in Mode 1 (*β* = 0.052, *p* > 0.05) and Mode 2 (*β* = 0.074, *p* > 0.05) are unsupported, but those in Mode 3 (*β* = 0.196, *p* < 0.001) are supported.
(6) It can be seen from the above results that the path coefficient of the model is different among the three pro-environmental publicity modes. Hypothesis 6 is supported.

## 5. Discussion

### 5.1. Attitude

Figure 3 shows that the attitude score in the centralized pro-environmental publicity mode is significantly higher than that in the one-on-one and face-to-face pro-environmental publicity and no-intervention modes. The attitude score in the one-on-one and face-to-face pro-environmental publicity mode was higher than that in the no-intervention mode. The analysis of the multi-group structural equation model (Figure 4) shows that the influence of attitude on intention is significantly different under the three different publicity modes. First, the centralized pro-environmental publicity mode can improve the attitude of farmers toward straw recycling to the largest extent, but the attitude has no significant effect on intention, which has similar results to other studies on farmer participation in community governance [66]. Farmers were gathered to popularize the knowledge of straw-burning prohibition and recycling in the form of meetings, and the group effect was activated. Therefore, the recycling intention of farmers is more easily affected by the atmosphere compared with their recycling attitude. Second, a collective atmosphere was not observed in the non-intervention mode and the one-on-one and face-to-face pro-environmental publicity mode, and the recycling intention of farmers is consistent with their attitude.

### 5.2. Subjective Norms

Figure 3 shows that subjective norms have the highest score under the centralized pro-environmental publicity mode, followed by the one-on-one and face-to-face pro-environmental publicity mode. Figure 4 reveals that subjective norms in the non-intervention mode have no significant effect on intention, which contradicts the original TPB model and previous studies [28]. Rural areas are a society of acquaintances, and the intentions of farmers to recycle straw are easily influenced by the people around them. However, the field investigation found that the local pro-environmental publicity on straw recycling was weak, and many farmers did not consider recycling the straw. Moreover, most farmers return their straw to the field or throw them away. The promoting effect of subjective norms on recycling intention will be weakened when behavior that is not conducive to straw recycling becomes prevalent in the village [67]. The subjective norms in the one-on-one and face-to-face environmental publicity mode and the centralized environmental publicity mode have a significant positive effect on intention, which is consistent with the results of other studies on environmental governance [68]. This finding may be due to the comprehensive understanding of farmers of straw recycling after receiving pro-environmental publicity from researchers. Moreover, concentrated propaganda is to gather “a group of acquaintances”, which is conducive to promoting the effect of subjective norms on intentions.

### 5.3. Perceptual Behavioral Control

Figure 3 shows that the perceived behavior control scores in the one-on-one and face-to-face pro-environmental publicity mode and the no-intervention publicity mode are significantly higher than those in the centralized pro-environmental publicity mode. Thus, farmers believe that straw recycling will cost additional time and energy after receiving the focused pro-environmental publicity. Combined with Figure 4, the influence of perceived behavior control on intention is insignificant in the three different pro-environmental publicity modes, which is inconsistent with the related studies on straw recycling [69]. Straw recycling is a relatively new concept for local people, and local people lack the dissemination of knowledge regarding straw recycling in daily publicity, which will inhibit the influence of perceived behavioral control on recycling intention. Furthermore, the imperfections of past behavior and straw recycling facilities will weaken the influence of perceived behavioral control on straw recycling intention [68,70]. Interestingly, the perceived behavioral control scores decreased when the farmers received concentrated and one-on-one pro-environmental publicity. Enhanced learning of straw recycling knowledge of farmers can generally promote their perceptual behavioral control [71]. However, farmers must collect straw by themselves and transport it to a distant recycling station due to the lack of local straw recycling facilities, thus contributing to the dilemma of farmers after receiving relevant education. By contrast, the emergence of new technology or concept, especially when accompanied by changes in the previous behavior habits, will also weaken the perceived behavioral control of farmers.

### 5.4. Social Trust

Figure 3 shows no significant difference in social trust scores among the three pro-environmental publicity modes. Figure 4 indicates that social trust under the three pro-environmental publicity modes has a significant positive effect on straw recycling intention. The research results of agricultural waste recycling [22], participation of farmers in community governance [42], and household recycling behavior [45] are consistent with the current study despite the absence of studies that demonstrate the effect of social trust on straw recycling intention of farmers. As a mechanism to simplify complexity, social trust optimizes relationships among people, reduces social transaction costs, enhances the value identification of collective action cooperation, and provides the motivation for farmers to participate in straw recycling. Interestingly, social trust was the factor with the highest degree of influence on intention between the one-on-one and face-to-face and centralized pro-environmental publicity modes. Improving the social trust of farmers in rural areas with relatively backward economic development levels is an effective factor to promote pro-environmental consciousness and implement the strategy of sustainable development.

### 5.5. Value Perception

Figure 3 shows that the value perception score in the centralized pro-environmental publicity mode is significantly higher than that in the two other pro-environmental publicity modes. Although concentrated pro-environmental publicity can effectively help farmers realize the value of straw recycling, value perception under the concentrated pro-environmental publicity mode has no significant influence on straw recycling intention (Figure 4). This “disconnect between value perception and intention” is mainly due to the group effect. Farmers are markedly affected by the collective atmosphere, which weakens the influence of value perception on recycling intention. However, the value perception in the non-intervention mode also has no significant effect on intention. This finding may be due to the imperfect local recycling facilities and the lack of knowledge dissemination of the straw recycling value, convincing farmers that the cost perception of straw recycling exceeds their value. The high perceived cost of farmers contributes to their limited willingness to participate in the utilization of agricultural waste resources. The survey also found that some farmers were even willing to give straw away for free if a recycling agency came to collect straw. The value perception of farmers under the one-on-one and face-to-face pro-environmental publicity mode has a significant positive effect on recycling intention. This result indicates that the pro-environmental publicity of straw recycling can promote the impact of value perception on the straw recycling intention by removing the collective atmosphere of the centralized pro-environmental publicity mode.

### 5.6. Theoretical Significance

From the perspective of social psychology, this study incorporated social trust and value perception into the TPB, explored the driving factors of farmers’ intentions to participate in straw recycling and deepened the understanding of the mechanism of crop straw recycling behavior from the existing research. Existing studies have confirmed that pro-environmental publicity has a significant impact on the waste recycling behavior of residents. However, studies that considered the psychological changes of farmers among different pro-environmental publicity modes are few. This study compared the differences in the influence of driving factors of farmers’ straw recycling intentions among three specific pro-environmental publicity modes and enriched the theoretical system of the straw recycling intentions of farmers.

### 5.7. Management Implications

With the continuous consumption of non-renewable energy and the increasingly prominent environmental problems, improving the straw recycling intentions of farmers is of considerable practical significance. This study explored the influencing factors of the straw recycling intentions of farmers and their influencing mechanisms based on the three different pro-environmental publicity modes and proposed the following suggestions.

#### 5.7.1. Organic Combination of Different Pro-Environmental Publicity Modes

The centralized pro-environmental publicity mode can maximize the improvement of attitudes, subjective norms, value perceptions, and recycling intentions of farmers. The one-on-one and face-to-face pro-environmental publicity mode can effectively promote the influence of attitudes, subjective norms, and value perception on recycling intention. Therefore, centralized publicity and one-on-one face-to-face publicity are integrated on the basis of non-intervention mode to maximize their respective advantages. Local governments can invite volunteers to the countryside during university holidays to conduct one-on-one advocacy for farmers. The volunteers can be given awards or certificates after the publicity activities to achieve a win–win situation between science popularization of farmers and social practice of students. In addition, village cadres could organize farmers to study intensively in the slack season to improve their straw recycling intention.

#### 5.7.2. Improve Recycling Facilities to Reduce Straw Recycling Difficulty

The imperfection of recycling facilities not only reduces the effect of perceived behavior control on intention but also minimizes the effect of perceived value on intention. The local government should set up a temporary collection point in the area with additional straw during the harvest season to lessen the time and energy consumption of farmers in the process of straw recycling and transporting. In addition, the government can cooperate with straw recycling agencies to protect the local environment while also increasing the income of farmers.

#### 5.7.3. Strengthen the Cultivation of Social Trust to Enhance Straw Recycling Enthusiasm

This study validates and emphasizes the importance of social trust in the straw recycling intention of farmers. Therefore, the government and the community should focus on the impact of social trust while strengthening pro-environmental publicity and education and improving infrastructure. Social trust is not given in advance but must be actively built by each farmer. First, the building of institutional trust should be strengthened. The government attaches considerable importance to the construction of service-oriented governments, thus standardizing the formulation of straw recycling policies, perfecting the supervision mechanism of policy implementation, and strengthening the transparency of policy implementation information to improve the institutional trust level of farmers. Second, the construction of interpersonal trust should also be strengthened. The government can foster a social atmosphere of mutual trust and benefit through the media. Community collective activities can be held frequently to promote contact between farmers and strengthen their interpersonal trust.

## 6. Conclusions

Social trust and value perception were incorporated into the TPB in this study, and a driving factor model of the straw recycling intentions of farmers was constructed [63]. The influence mechanism of attitude, subjective norms, perceived behavior control, social trust, and value perception on straw recycling intention was discussed among the three pro-environmental publicity modes. This study fills a research gap on the relationship between social trust and straw recycling intention and explores the effect of specific pro-environmental publicity on straw recycling intentions. The main conclusions of this study are as follows.

(1) The concentrated pro-environmental publicity mode has the most significant effect on improving the attitude, subjective norms, value perception, and recycling intentions of farmers towards straw recycling.
(2) Social trust is a key factor in determining the straw recycling intentions of farmers. The intention of farmers to participate in straw recycling is strengthened when the social trust degree is high.
(3) Attitude, subjective norms, and value perception are important factors for farmers to participate in straw recycling. Significant differences were observed in the effects of attitudes, subjective norms, and value perception on the straw recycling intentions of farmers among different pro-environmental publicity modes.

The above conclusions confirm the significant influence of social trust and value perception on the straw recycling intentions of farmers. The results also confirmed the difference in the influence of different pro-environmental publicity modes on the straw recycling intentions of farmers, which enriched the theoretical system of driving factors of their straw recycling intention. The following suggestions are proposed: (1) organic combination of different publicity modes, (2) improvement of recycling facilities to reduce straw recycling difficulty among farmers, and (3) strengthening the cultivation of social trust to enhance the straw recycling enthusiasm of farmers.

Despite the above contributions, this study also has the following limitations. (1) This study explored the driving factors of the straw recycling intention of farmers from the perspective of social psychology. The specific relationship between intention and behavior could not be explored without considering the specific recycling behavior. (2) Six sample villages in Chengdu were taken as research samples in this study, and the generality of the constructed straw recycling intention drivers was uncertain. (3) This study adopts cross-section data, ignoring the influence of time dimension on the psychology and straw recycling intention of farmers. Further consideration of panel data is needed in future studies.

## Figures and Tables

**Table 1:** Item measurements.

Variable	Item	Content	Factor Load	Mean
A	A1	I think straw recycling will contribute to energy saving and environmental protection.	0.788	3.791
	A2	I think straw recycling will improve the living environment.	0.804	3.777
	A3	Straw recycling can create a good environment for future generations.	0.861	3.765
	A4	Straw thrown away will pollute the environment.	0.873	3.817
	A5	Improper handling of straws will affect human health.	0.817	3.763
SNs	SNs1	I will participate in straw recycling because my neighbors and friends also do the same.	0.867	3.514
	SNs2	Many people suggested that I choose the straw recycling method.	0.768	2.602
	SNs3	Everyone supports my participation in straw recycling.	0.830	2.909
PBC	PBC1	Straw recycling will take me a considerable amount of time.	0.971	3.202
	PBC2	Straw recycling will cost me a considerable amount of energy.	0.980	3.198
VP	VP1	Straw recycling can gain economic benefits.	0.870	3.191
	VP2	Straw has recycling value.	0.548	3.495
	VP3	Straw recycling is conducive to saving resources and turning waste into wealth.	0.781	3.553
ST	ST1	I trust my relatives.	0.608	4.054
	ST2	I trust my neighbors.	0.864	3.947
	ST3	I trust the village officials.	0.866	3.900
	ST4	I believe in environmental laws.	0.880	3.995
IN	IN1	I am willing to recycle straw in my future life.	0.918	3.896
	IN2	I am willing to cooperate with the recycling staff to recycle straw.	0.918	3.920
	IN3	I am willing to transport the straw for recycling in the case of a short distance.	0.718	3.413

**Table 2:** Percentage of sampling.

	Sample Village	Village Group	Permanent Population	Effective Sample Size	Effective Sampling Proportion
Hilly area	Hongqi Village	23	3405	160	4.70%
	Shuangxin Village	17	1913	129	6.74%
Construction area	Zhongfu Community	14	3048	181	5.94%
	Shuangjiang Community	11	4938	94	1.90%
Plain area	Ronghua Village	31	4960	126	2.54%
	Shuangyan Village	23	5812	96	1.65%

**Table 3:** Distribution of the socio-demographic characteristics of the samples.

Profile	*N*	%	Profile	*N*	%
Gender			Family annual income		
Male	354	46.5	Below 20,000	228	30
Female	407	53.5	20,000–39,999	320	42
Age			40,000–59,999	106	14
Under 30	24	3.2	60,000 and above	107	14
30–39	65	8.5	Education level		
40–49	181	23.8	Not been to school	124	16.3
50–59	240	31.5	Primary school	341	44.8
60–69	156	20.5	Junior high school	219	28.8
70 and above	95	12.5	Senior high school	53	7
			University and above	24	3.2

**Table 4:** Reliability and validity tests.

Construct	CA ^a^	CR ^b^	AVE ^c^	A	IN	PBC	SNs	ST	VP
A	0.886	0.917	0.688	0.829 ^d^					
IN	0.811	0.891	0.733	0.366	0.856				
PBC	0.949	0.975	0.951	0.067	−0.033	0.975			
SNs	0.783	0.862	0.677	0.254	0.295	0.225	0.823		
ST	0.830	0.884	0.660	0.19	0.318	−0.088	0.154	0.812	
VP	0.630	0.784	0.556	0.464	0.312	0.085	0.511	0.025	0.746

Note: ^a^ CA: Cronbach’s alpha. ^b^ CR: Composite Reliability. ^c^ AVE: Average variance extracted. ^d^ The bold value on the diagonal line is AVE, and the value on the off-diagonal line is the correlation between the constructs.

## Data Availability

The data are available from the corresponding author upon reasonable request.
